# Gender differences in presentation and diagnosis of chest pain in primary care

**DOI:** 10.1186/1471-2296-10-79

**Published:** 2009-12-14

**Authors:** Stefan Bösner, Jörg Haasenritter, Maren A Hani, Heidi Keller, Andreas C Sönnichsen, Konstantinos Karatolios, Juergen R Schaefer, Erika Baum, Norbert Donner-Banzhoff

**Affiliations:** 1 Department of General Practice/Family Medicine, University of Marburg, 35032 Marburg, Germany; 2 Department of Family Medicine, Paracelsus University, 5020 Salzburg, Austria; 3 Department of Cardiology, University of Marburg, 35032 Marburg, Germany

## Abstract

**Background:**

Chest pain is a common complaint and reason for consultation in primary care. Research related to gender differences in regard to Coronary Heart Disease (CHD) has been mainly conducted in hospital but not in primary care settings. We aimed to analyse gender differences in aetiology and clinical characteristics of chest pain and to provide gender related symptoms and signs associated with CHD.

**Methods:**

We included 1212 consecutive patients with chest pain aged 35 years and older attending 74 general practitioners (GPs). GPs recorded symptoms and findings of each patient and provided follow up information. An independent interdisciplinary reference panel reviewed clinical data of every patient and decided about the aetiology of chest pain at the time of patient recruitment. Multivariable regression analysis was performed to identify clinical predictors that help to rule in or out CHD in women and men.

**Results:**

Women showed more psychogenic disorders (women 11,2%, men 7.3%, p = 0.02), men suffered more from CHD (women 13.0%, men 17.2%, p = 0.04), trauma (women 1.8%, men 5.1%, p < 0.001) and pneumonia/pleurisy (women 1.3%, men 3.0%, p = 0.04) Men showed significantly more often chest pain localised on the right side of the chest (women 9.1%, men 25.0%, p = 0.01). For both genders known clinical vascular disease, pain worse with exercise and age were associated positively with CHD. In women pain duration above one hour was associated positively with CHD, while shorter pain durations showed an association with CHD in men. In women negative associations were found for stinging pain and in men for pain depending on inspiration and localised muscle tension.

**Conclusions:**

We found gender differences in regard to aetiology, selected clinical characteristics and association of symptoms and signs with CHD in patients presenting with chest pain in a primary care setting. Further research is necessary to elucidate whether these differences would support recommendations for different diagnostic approaches for CHD according to a patient's gender.

## Background

Chest pain is a common complaint and reason for consultation in primary care and incidence varies according to setting, country and inclusion criteria [1-3] Chest pain can be caused by a wide range of different diseases including Coronary Heart Disease (CHD)[4,5] Extensive research has been conducted related to gender differences in regard to CHD [6-8] and to clinical characteristics in patients with Acute Coronary Syndrome (ACS) or Myocardial Infarction (MI) [9-11]. However, most of this research has been performed in emergency departments and data from a primary care context are lacking.

To our knowledge this is the first prospective primary care study investigating the epidemiology of chest pain where the large sample size allows a statistical analysis for gender differences. We aimed to analyse gender differences in aetiology, clinical characteristics, risk factors and comorbidities of chest pain in a primary care setting. In addition we wanted to provide gender related symptoms and signs that could support rational diagnosis of CHD.

## Methods

We conducted a cross-sectional diagnostic study with a delayed-type reference standard in a primary care setting[12] The final diagnosis was established by an expert panel after 6 months of follow up. The main aim of the study was to investigate the diagnostic accuracy of signs and symptoms for chest pain patients with CHD. In this article we report results in regard to gender differences in chest pain patients with a focus on CHD as the final primary outcome.

### Participating GPs and patients

We used an existing network of collaborating research practices to approach 209 GPs in the State of Hesse of whom 35.4% agreed to participate in the study. Only GPs being prepared to undergo random recruitment audits could take part. The 74 participating doctors (58 practices) had to recruit consecutively every attending patient with chest pain, both as presenting complaint or on questioning. The recruitment period lasted 12 weeks for each practice and was staggered in four waves between October 2005 and July 2006. Informed consent was obtained from all patients and documented accordingly.

Every patient above 35 years with pain localized in the area between clavicles and lower costal margins and anterior to the posterior axillary lines had to be included. Doctors were also asked to recruit during home visits and emergency calls. Patients were eligible irrespective of the acute or chronic nature of their complaints, of previously known conditions including CHD or related risk factors. Patients whose chest pain had subsided for more than one month, whose chest pain had been investigated already and/or who came for follow-up for previously diagnosed chest pain were excluded. Like the whole study protocol, this procedure was approved by the Ethics Committee of the Faculty of Medicine, University of Marburg. The study complies with the declaration of Helsinki.

### Data collection

#### Baseline

GPs took a standardized history and performed a physical examination according to a case report form (CRF) that was piloted and modified accordingly. The CRF covered information on basic patient and pain characteristics, accompanying symptoms and risk factors for CHD. GPs also recorded their preliminary diagnoses, investigations and management related to the patients' chest pains.

#### Follow up

Patients were contacted by phone six weeks and six months after the index consultation. Study assistants, who were blinded to clinical data previously recorded, asked about the course of the patients' chest pain, treatments including hospitalisations and drugs. Discharge letters from specialists and hospitals were requested by GPs.

#### Diagnosis and reference standard

A reference panel consisting of one cardiologist, one GP and one research associate (also a trained GP) of the department of Family Medicine reviewed baseline and follow up data of each patient. They decided on the most likely medical condition having caused the individual patient's chest pain at the time of the index test (delayed type reference standard). The GP's initial diagnosis contributed to the decision made by the panel.

### Statistical analysis

The analyses for gender differences in regard to the final diagnoses and the probabilities for any CHD are based on the sample of all patients with chest pain where diagnostic classification was possible. Clinical characteristics, risk factors and comorbidities were analysed for patients with CHD as final diagnosis.

For univariate analyses we calculated proportions and diagnostic odds ratios (OR) for all clinical items covered by the CRF. The Chi-Square test was used for univariate comparisons of categorical data. Fisher's exact test was used when the nominator was equal or below five. We calculated the z-ratio and associated two-tail probabilities for the difference between proportions. As this is an explorative study including many comparisons between different variables, p < 0.01 was considered to provide evidence of an association, while p < 0.05 was considered to indicate a possible association[13] Index test items that had a p-value < 0.1 were included as independent variables in multivariate logistic regression analysis. The dependent variable was CHD. Variable selection (removal) was conducted using the backward stepwise procedure (p < 0.05). Odds ratio and 95%-confidence intervals were calculated. Likelihood Ratios (LR) of significant predictors were calculated based on univariate data (4 × 4 tables). Analyses were performed with SPSS software version 15.0.

## Results

### GP and patient characteristics and results of random audits

**Figure 1:**
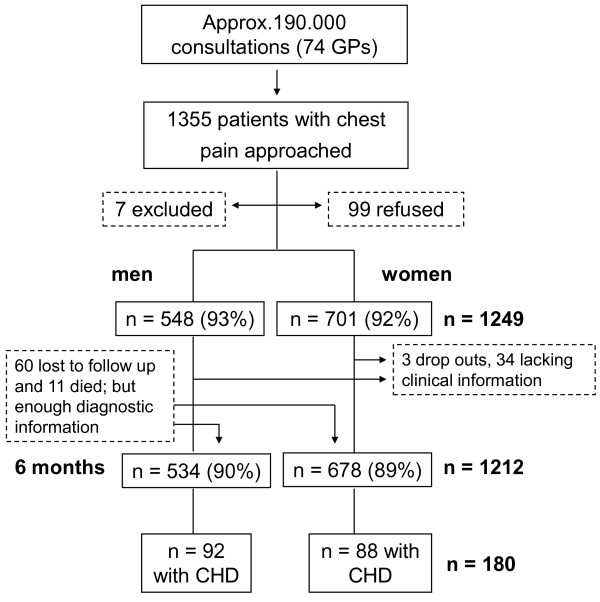
**Patient Flow**.

The majority of the participating 74 GPs were male (67%), mean age of GPs was 49 years. Two thirds of the practices were located in urban areas (63.5%). The participating GPs' demographic characteristics are similar to the population of GPs in the State of Hesse. According to our estimate participating GPs encountered around 190.000 patients during the study period and approached 1355 patients with chest pain. 7 patients did not meet the inclusion criteria and 99 refused to participate in the study. GPs returned valid CRFs (T0) for 1249 patients (548 men and 701 women). 60 cases were consequently lost to follow up and 11 died but provided enough information to be judged by the reference committee; 3 cases were early drop outs (2 women and 1 man) and were therefore not included. For 34 cases (22 women and 12 men) follow up information was lacking, incomplete or ambiguous so that no final diagnosis could be made (3.1% of women and 2.2% of men with a valid case report form). At T1 (6 months) we thus analysed 1212 patients (534 men and 678 women) for the aetiology of their chest pain; of those 180 patients (92 men and 88 women) were diagnosed as having CHD (figure 1).

We conducted 68 random audits. Of the 68 recruitment days analysed, 54 GPs did include all patients, 8 GPs forgot to include 1 patient, 3 GPs forgot to include 2 patients and 2 GPs forgot to include 3 patients. For all the missed patients the CRF could still be completed.

Table 1 provides basic characteristics of the study population. Overall more women were consulting their GP. In both groups, women and men, the vast majority of patients were known by their GP from former consultations (with women presenting a significantly higher proportion), most patients were quoting chest pain as reason for the actual consultation, nearly half of the patients had acute chest pain at the time of consultation, around 60% assumed a cardiac origin of their chest pain and nearly one third of patients presented themselves with acute chest pain. There was an almost identical age distribution.

**Table 1:** Basic characteristics of the study population (all patients with chest pain, n = 1249)

Patient characteristics	**Women **(n = 701)	**Men **(n = 548)	p-value
Age in age groups			
< = 44 - n (%)	119 (17.0)	117 (21.4)	0.05
45-54 - n (%)	143 (20.4)	115 (21.0)	0.8
55-64 - n (%)	128 (18.3)	115 (21.0)	0.23
65-74 - n (%)	179 (25.5)	128 (23.4)	0.38
> = 75 - n (%)	132 (18.8)	73 (13.3)	**< 0.001**

Patients having chest pain at the time of consultation - n (%)*	325 (47.0)	243 (45.3)	0.48

Patients known to GP - n (%)*	656 (93.7)	492 (90.3)	**0.03**

Patient assumes cardiac origin of chest pain - n(%)	386 (62.7)	288 (58.3)	0.38

Chest pain as reason for consultation - n (%)*	619 (88.4)	473 (86.6)	0.29

Acute pain (<48 hrs, including 14 trauma cases) - n (%)*	198 (28.8)	166 (30.7)	0.43

* slightly changing denominator because of missing data

### Aetiology of chest pain

Chest wall syndrome (CWS), CHD, psychogenic disorders and upper respiratory infections constituted the main diagnostic groups for both genders making up 80.2% of all diagnoses in women and 77.5% in men. Women showed significantly more psychogenic disorders, men suffered more from CHD, trauma and pneumonia and/or pleurisy (see table 2).

**Table 2:** Final diagnoses in patients presenting with chest pain to their GP (all chest pain patients where a final diagnosis could be established, n = 1212)

Diagnosis	Women (n = 678)	Men (n = 534)	p-value
Chest wall syndrome	330 (48.6%)	235 (44.0%)	0.14

CHD (stable **and **ACS)	88 (13.0%)	92 (17.2%)	**0.04**

CHD (stable)	68 (10.0%)	68 (12.7%)	0.11

Psychogenic disorders	76 (11.2%)	39 (7.3%)	**0.02**

Upper respiratory infections	50 (7.4%)	48 (9.0%)	0.29

Acute coronary syndrome (ACS)	20 (2.9%)	24 (4.5%)	0.15

Gastroesophageal reflux disease	25 (3.7%)	17 (3.2%)	0.65

Trauma	12 (1.8%)	27 (5.1%)	**<0.001**

Benign stomach problems	19 (2.8%)	7 (1.3%)	0.08

Pneumonia and/or pleurisy	9 (1.3%)	16 (3.0%)	**0.04**

COPD/Asthma	10 (1.5%)	13 (2.4%)	0.22

Other	59 (8.7%)	40 (7.5%)	0.45

### Clinical characteristics

Table 3 shows clinical characteristics of chest pain patients for CHD stratified by gender. While significantly more women had chest pain lasting from 1-12 hours, more men were found to have pain lasting from 30-60 minutes. In both groups most patients had pain duration between 1-30 minutes. There were no gender differences in the frequency and time of onset of pain with more than half of the patients having chest pain more than once a day. Pain character was described by the majority in both groups as 'pressure'. A possible association was found for more men reporting burning, and more women reporting dull pain. A respiratory infection was found more often in women as accompanying symptom. Pain was localised in two thirds of both gender groups on the left side of the chest. However, men showed significantly more often chest pain localised on the right side of the chest. Significant differences also existed for the findings of the physical examination with women showing more localised muscle tension.

**Table 3:** Clinical characteristics of chest pain patients for any CHD (acute or chronic) by gender (only patients with CHD as final diagnosis, n = 180)

	Clinical characteristics	Women (n = 88)*	Men (n = 92)*	OR (95%-CI)	p
Presentation and duration of a pain episode	Pain at time of consultation	61 (82.4%)	71 (85.5%)	0.79 (0.34-1.87)	0.48
	Continuous pain	9 (10.2%)	8 (8.7%)	1.20 (0.44-3.25)	0.73
	12-24 hrs	4 (4.5%)	3 (3.3%)	1.41 (0.31-6.50)	0.66
	**1-12 hrs**	**21 (23.9%)**	**8 (8.7%)**	**3.29 (1.37-7.90)**	**<0.01**
	**30-60 min**	**9 (10.2%)**	**21 (22.8%)**	**0.39 (0.17-0.90)**	**0.02**
	1-30 min	32 (36.4%)	42 (45.7%)	0.68 (0.37-1.24)	0.21
	< 1 min	12 (13.6%)	10 (10.9%)	1.30 (0.53-3.17)	0.57

Frequency of pain	More than once a day	43 (51.8%)	48 (55.2%)	0.87 (0.48-1.60)	0.66
	Once a day	17 (20.5%)	13 (14.9%)	1.47 (0.66-3.25)	0.34
	Less frequently than once a day	18 (21.7%)	21 (24.1%)	0.87 (0.43-1.78)	0.70

Time of onset of pain	Early morning	2 (2.5%)	4 (4.7%)	0.53 (0.09-2.95)	0.46
	Morning	4 (5.0%)	2 (2.3%)	2.21 (0.39-12.41)	0.36
	Midday	8 (10.0%)	3 (3.5%)	3.07 (0.79-12.02)	0.09
	Evening	4 (5.0%)	3 (3.5%)	1.46 (0.32-6.72)	0.63
	Night	10 (12.5%)	6 (7.0%)	1.91 (0.66-5.51)	0.23

Pain character	Pressure	53 (60.9%)	58 (64.4%)	0.86 (0.47-1.58)	0.63
	**Burning**	**4 (4.6%)**	**13 (14.4%)**	**0.29 (0.09-0.91)**	**0.03**
	Stinging	14 (16.1%)	21 (23.3%)	0.63 (0.30-1.34)	0.23
	**Dull**	**25 (28.7%)**	**14 (15.6%)**	**2.19 (1.05-4.57)**	**0.03**

Other symptoms	Nausea/vomiting	5 (5.7%)	4 (4.3%)	1.33 (0.34-5.11)	0.68
	Dyspnoea	27 (30.7%)	34 (37.0%)	0.76 (0.41-1.40)	0.37
	Tightness	48 (54.5%)	43 (46.7%)	1.37 (0.76-2.46)	0.30
	Cough	4 (4.5%)	3 (3.3%)	1.41 (0.31-6.50)	0.66
	**Respiratory infection**	**7 (8.0%)**	**1 (1.1%)**	**7.86 (0.95-65.29)**	**0.03**

Pain depending on	Exercise	34 (38.6%)	44 (47.8%)	0.69 (0.38-1.24)	0.21
	Inspiration	5 (5.7%)	4 (4.3%)	1.33 (0.34-5.12)	0.68
	Movement	14 (15.9%)	16 (17.4%)	0.90 (0.41-1.97)	0.79
	Food intake	0 (0%)	1 (1.1%)	0.51 (0.44-0.59)	0.33

Localisation of pain	Retrosternal	7 (8.0%)	6 (6.5%)	1.24 (0.40-3.84)	0.22
	Left side of chest	56 (63.6%)	63 (68.5%)	0.81 (0.43-1.50)	0.49
	**Right side of chest**	**8 (9.1%)**	**23 (25.0%)**	**0.30 (0.17-0.71)**	**0.01**
	Upper abdomen	10 (11.4%)	5 (5.4%)	2.23 (0.73-6.81)	0.15

Radiation of pain	Left side of chest	16 (18.2%)	14 (15.2%)	1.24 (0.56-2.72)	0.59
	Left arm	14 (15.9%)	13 (14.1%)	1.15 (0.51-2.61)	0.74
	Right side of chest	6 (6.8%)	3 (3.3%)	2.17 (0.53-8.96)	0.27
	Right arm	6 (6.8%)	3 (3.3%)	2.17 (0.53-8.96)	0.07
	Abdomen	3 (3.4%)	4 (4.3%)	0.78 (0.17-3.57)	0.75
	Retrosternal	7 (8.0%)	6 (6.5%)	1.24 (0.40-3.84)	0.71
	Back	10 (11.4%)	13 (14.1%)	0.78 (0.32-1.88)	0.58

Physical examination	**Localised muscle tension**	**20 (32.3%)**	**7 (9.1%)**	**4.76 (1.86-12.21)**	**0.001**
	Pain reproducible by palpation	10 (18.5%)	4 (6.3%)	3.35 (0.99-11.39)	0.05

*numbers vary slightly because of missing index test data

**Table 4:** Clinical characteristics of all chest pain cases associated with CHD by gender (multivariable model, n = 1210)

Index test	Women (n = 689)		Men (n = 521)	
	
	adjusted OR (95%-CI)	p value	adjusted OR (95%-CI)	p value
Known clinical vascular disease*	5.71 (0.16-1.11)	0.001	19.56 (5.03-76.03)	<0.001

Pain worse with exercise	3.59 (1.24-10.39)	0.019	4.43 (1.32-14.86)	0.016

Age/gender (female ≥ 65, male ≥ 55)	3.77 (1.26-11.32)	0.018	7.78 (2.05-29.57)	0.003

Stinging pain	0.30 (0.09-0.96)	0.042		

Diabetes mellitus	3.82 (1.40-10.39)	0.009		

Pain duration between 1-12 hours	3.96 (1.52-10.33)	0.005		

Patient assumes cardiac origin of pain			8.26 (1.77-38.67)	0.007

Pressing pain			3.60 (1.16-11.22)	0.027

Pain depending on inspiration			0.07 (0.01-0.65)	0.019

Localized muscle tension			0.12 (0.03-0.44)	0.001

Pain radiating to the back			11.29 (1.93-66.10)	0.007

Pain duration between 30 min.-1 hour			5.75 (1.32-25.09)	0.020

Pain duration between 1-30 min.			4.27 (1.25-14.66)	0.021

The following variables were selected for multivariable analysis (according to performance markers in univariate analysis: p < 0.1): **For men**: patient assumes cardiac origin of pain, GP's impression that something is wrong with the patient, cold sweat, chest pain at time of consultation, pressing pain, stinging pain, dyspnoea, tightness, cough, respiratory infection, pain worse with breathing, pain worse with movement, heart failure, hyperlipidemia, diabetes mellitus, hypertension, localised muscle tension, pain reproducible by palpation, epigastric pain, pain radiating to the back, pain radiating to the left side, continuous pain, pain duration between 30 min.-1 hour, pain duration between 1-30 min, pain less than once a day, pain in the evening, known clinical vascular disease, pain depending on exercise, age ≥ 55. **For women**: patient assumes cardiac origin of pain, GP's impression that something is wrong with the patient, chest pain at time of consultation, pressing pain, stinging pain, dyspnoea, tightness, cough, pain worse with breathing, pain worse with movement, heart failure, hyperlipidemia, diabetes mellitus, hypertension, localised muscle tension, pain reproducible by palpation, continuous pain, pain duration between 1-30 min, pain in the evening, known clinical vascular disease, pain depending on exercise, age ≥ 65, chest pain as reason for consultation, pallor, burning pain, dull pain, pain on ingestion, smoking, lack of activity, pain on right side, pain duration 1-12 hours, retrosternal location, pain at noon time. * criterion positive if CHD or occlusive vascular disease or cerebrovascular disease

### Risk factors and comorbidities

Except for smoking status which was significantly higher for men (men 14.1%, women 3.4%, p = 0.03) there were no gender related differences in other risk factors (hyperlipidemia, diabetes mellitus, hypertension, overweight and family history of CHD/Myocardial Infarction) for chest pain patients with any CHD (acute or chronic).

No significant gender differences were observed for cerebrovascular disease, heart failure, peripheral occlusive arterial disease, diabetes mellitus and hypertension as comorbidities in chest pain patients with CHD.

### Association of clinical characteristics with CHD - univariate and multivariate analysis

33 items for women and 29 items for men (listed in the footnote of table 4) fulfilled our univariate selection criteria and were selected for multivariable analysis. The results are reported in table 4.

For both genders known clinical vascular disease, pain worse with exercise and age were associated positively with CHD. In women pain duration above one hour was associated positively with CHD, while shorter pain durations showed an association with CHD in men. In women negative associations were found for stinging pain and in men for pain depending on inspiration and localised muscle tension.

Table 5 presents the corresponding Likelihood ratios (LR) for CHD for the above mentioned findings being absent or present.

**Table 5:** Clinical recommendation

Useful for in- or excluding any CHD in women	Likelihood Ratio if Finding	
	
	Present	Absent
Known clinical vascular disease	5.52 (3.97; 7.67)	0.55 (0.45; 0.68)

Pain worse with exercise	2.21 (1.61; 3.04)	0.74 (0.63; 0.88)

Age ≥ 65 years	2.04 (1.77; 2.37)	0.34 (0.22; 0.51)

Stinging pain	0.41 (0.25; 0.67)	1.39 (1.24; 1.55)

Diabetes mellitus	2.35 (1.58; 3.50)	0.81 (0.71; 0.93)

Pain duration between 1-12 hours	1.65 (1.09; 2.52)	0.89 (0.79; 1.01)

**Useful for in- or excluding any CHD** **in men**	**Likelihood Ratio if Finding**	
	
	**Present**	**Absent**

Known clinical vascular disease	3.61 (2.70; 4.82)	0.53 (0.42; 0.66)

Pain worse with exercise	2.89 (2.14; 3.91)	0.63 (0.51; 0.76)

Age ≥ 55 years	1.64 (1.44; 1.86)	0.32 (0.19; 0.52)

Patient assumes cardiac origin of pain	1.55 (1.36; 1.76)	0.32 (0.19; 0.55)

Pressing pain	1.71 (1.40; 2.08)	0.57 (0.43; 0.76)

Pain depending on inspiration	0.17 (0.07; 0.47)	1.27 (1.18; 1.36)

Localized muscle tension	0.23 (0.11-0.47)	1.51 (1.35; 1.69)

Pain radiating to the back	1.66 (0.92; 3.00)	0.94 (0.86; 1.03)

Pain duration between 30 min.-1 hour	1.67 (1.07; 2.60)	0.89 (0.80; 1.01)

Pain duration between 1-30 min.	1.79 (1.36; 2.36)	0.73 (0.60; 0.89)

## Discussion

The aim of our study was the description of gender differences in patients presenting with chest pain in a primary care setting with a special focus on the subgroup of patients who were diagnosed with CHD. More women than men were suffering from chest pain. We found gender differences for several aetiologies of chest pain. The subgroup of CHD patients did not show gender differences in regard to most clinical characteristics.

To our knowledge this is the first prospective study with a sufficient sample size that allows investigating gender differences of chest pain in primary care. Strengths of our study are a large GP based consecutive sample which is highly representative, a prospective design and small drop out rates. Study procedures, including random audits, reduced the possibility of selection bias and an interdisciplinary team provided a precise diagnosis as reference standard.

We did not interfere with the work-up provided by participating GPs. As a result of this for some patients only limited clinical data were available to the reference panel. Since data from the original questionnaire including GPs' diagnoses were also used by the panel for decision making there may be a certain degree of incorporation bias in regard to the final diagnoses[14] Cases with incomplete data did not differ in mean age but comprised a higher percentage of women than the study sample which might have led to some bias.

Our sample contained a priori a significant higher number of women than men who presented with chest pain to their GP. While those data might reflect real gender differences in underlying aetiologies, e.g. the biology of different diseases that can cause chest pain, another more likely explanation could be differences in self perception and symptom reporting between men and women. Two studies found that women rated their pain as more intense[15,16] using more affective words[16] to describe their pain, both mechanisms that might contribute to a lower threshold to consult a GP for further investigations. This is supported by observations that women report more often bodily symptoms than men[17,18]

We found no gender differences for the patients' assumption that their chest pain would be of cardiac origin. Granot et al. describe that women presenting with unstable angina pectoris related their chest pain less to heart disease[15] However, our sample contained a broad variety of aetiologies with only a minority of patients with unstable angina.

In regard to the aetiology of chest pain more women were suffering from psychogenic disorders compared to men. These findings are supported by studies that show clear gender differences in depressive patients[19,20] A meta analysis about predictors of panic disorder among patients with chest pain revealed female sex as one of five variables[21] More trauma cases were found in men, a fact which can be explained with a higher occupational risk for accidents.

For the subpopulation of patients finally diagnosed with CHD we found gender differences for certain clinical symptoms and signs. More women had a pain episode of 1-12 hours in comparison to more men reporting a shorter pain episode (30-60 minutes). For pain character and other accompanying symptoms we found only possible associations. This is in contrast to several other publications where women reported more nausea, burning pain and shortness of breath[10,11,22-24] However, most of these studies were conducted in emergency departments including patients with Myocardial Infarction (MI) or Acute Coronary Syndrome (ACS). This difference in findings could be explained by the fact that our analysis included patients with acute as well as chronic CHD.

Our data add as a second interesting finding that in more men than women pain localisation was on the right side of the chest. This finding is only partly supported by studies conducted in high prevalence settings which report gender differences for jaw pain[7,23] but none for left or right arm pain[10]. Kosuge et al. report in addition a significant higher proportion of right shoulder pain in women with ST-segment elevation acute MI[25] Again the differences in study setting and patient selection would be the most likely explanation. Overall, for CHD patients most clinical characteristics did not show gender differences. Our findings therefore do not support CHD being more "typical" in men.

Except for smoking which showed a possible association with male gender we did not find gender related differences in risk factors for CHD and in related comorbidities. This is supported by data from Western countries where smoking prevalence was partly still higher among men[7], partly already comparable between men and women[6]. Risk factors like hypertension and hyperlipidemia are more prominent for men than women in the late 40- to early 50-year range; then their prevalence is higher in women[6]

In women pain duration above one hour was associated positively with CHD while shorter pain durations showed an association with CHD in men. Chun et al. found in a systematic review about the accuracy of bedside findings for diagnosing CHD a pain duration of over 30 minutes to be a strong negative predictor[26]. In men we found negative association for localised muscle tension. In a diagnostic meta-analysis determining the accuracy of 10 important signs and symptoms only chest wall tenderness on palpation largely ruled out acute MI or ACS[27] Most existing studies on the diagnostic accuracy of symptoms and signs for CHD are conducted in high prevalence settings, include ACS or MI as main outcome parameters and do not stratify the results for gender [28-30] The results should therefore be compared with caution.

Most of the corresponding LRs are in a range that goes along with a small to moderate change in disease likelihood, insufficient to rule in or out disease[31] However, the family doctor can regard each of his questions and clinical examination maneuvers as a separate diagnostic test. Based on the pretest probability (disease prevalence for a given setting) a combination and stepwise application of these different diagnostic steps leads often to reasonable post test probabilities. Using this so called Bayesian approach can guide decisions in regard to further work up[32]

## Conclusions

In summary we found gender differences in regard to aetiology, selected clinical characteristics and association of symptoms and signs with CHD in patients presenting with chest pain in a primary care setting. Further research is necessary to elucidate whether these differences would support recommendations for different diagnostic approaches for CHD according to a patient's gender.

## Competing interests

Conflict of Interests: JRS acts as scientific advisor for MSD and ESSEX.

All other authors do not declare any competing interests.

## Authors' contributions

NDB formulated the research question, designed the study and supervised its conduct together with ACS. NDB, EB, JH, ACS, MAH, HK, JRS, KK and SB were involved in acquisition, analysis and interpretation of data. SB drafted the article; NDB, EB, JH, ACS, MAH, HK, JRS and KK revised it critically. All authors approved the final manuscript.

## Pre-publication history

The pre-publication history for this paper can be accessed here:

http://www.biomedcentral.com/1471-2296/10/79/prepub
